# Prevalence, Molecular Landscape, and Clinical Impact of *DICER1* and *DGCR8* Mutated Follicular-Patterned Thyroid Nodules

**DOI:** 10.1210/clinem/dgae034

**Published:** 2024-01-22

**Authors:** Vincenzo Condello, Anello M Poma, Elisabetta Macerola, Paola Vignali, Johan O Paulsson, Jan Zedenius, Fulvio Basolo, C Christofer Juhlin

**Affiliations:** Department of Oncology-Pathology, Karolinska Institutet, 171 64 Stockholm, Sweden; Department of Surgical, Medical, Molecular Pathology and Critical Care Medicine, University of Pisa, 56126 Pisa, Italy; Department of Surgical, Medical, Molecular Pathology and Critical Care Medicine, University of Pisa, 56126 Pisa, Italy; Department of Surgical, Medical, Molecular Pathology and Critical Care Medicine, University of Pisa, 56126 Pisa, Italy; Department of Oncology-Pathology, Karolinska Institutet, 171 64 Stockholm, Sweden; Department of Molecular Medicine and Surgery, Karolinska Institutet, 171 64 Stockholm, Sweden; Department of Breast, Endocrine Tumors, and Sarcoma, Karolinska University Hospital, 171 64 Stockholm, Sweden; Department of Surgical, Medical, Molecular Pathology and Critical Care Medicine, University of Pisa, 56126 Pisa, Italy; Department of Oncology-Pathology, Karolinska Institutet, 171 64 Stockholm, Sweden; Department of Pathology and Cancer Diagnostics, Karolinska University Hospital, 171 64 Stockholm, Sweden

**Keywords:** *DICER1*, *DGCR8*, miRNA, thyroid nodules

## Abstract

**Background:**

Mutations in micro-RNA (miRNA) regulators *DICER1* and *DGCR8* have recently been uncovered, revealing a potential novel mechanism driving thyroid tumor development. However, the true frequency of these hotspot mutations in follicular-patterned thyroid tumors (FTs) and their relation to established driver gene events remain elusive.

**Methods:**

A total of 440 FTs from 2 institutions were interrogated for *DICER1, DGCR8,* and *RAS* family hotspot mutations using Sanger sequencing. Whole-exome sequencing was also performed to identify additional driver gene aberrations in *DICER1/DGCR8*-mutant cases. Subsets of cases were further analyzed using miRNA expression profiling, and key dysregulated miRNAs were validated as markers of *DICER1* mutations using quantitative RT-PCR analysis. The Cancer Genome Atlas (TCGA) database was also probed for *DICER1*/*DGCR8* mutations and miRNA dysregulation.

**Results:**

Fourteen (3.2%) and 4 (1%) FTs harbored *DICER1* and *DGCR8* hotspot mutations, respectively, in the combined cohort, and no cases with normal tissue available were found to exhibit a constitutional variant. Two *DGCR8*-mutant cases also harbored oncogenic *RAS* mutations. Whole-exome sequencing analysis did not identify additional driver gene events in *DICER1*/*DGCR8-*positive cases. Comprehensive miRNA expression profiling revealed a unique pattern of dysregulated miRNAs in *DICER1*/*DGCR8-*mutant cases compared with wild-type lesions. Moreover, *DICER1*-mutant cases showed a remarkable reduction of 5′ arm miRNAs, findings corroborated in the TCGA cohort.

**Conclusion:**

*DICER1* and *DGCR8* hotspot mutations are rare in unselected cohorts of FTs, and mutated cases exhibit a specific miRNA profile. Although *DGCR8* mutations may coexist with established *RAS* gene alterations, FTs with *DICER1* variants were devoid of other driver gene events.

In the past several decades, the relevance of micro-RNA (miRNA) processing machinery genes has taken center stage in molecular pathology and oncology. Specific gene mutations have been identified in different human cancers, suggesting that aberrations in miRNA expression driven by these mutations might be an important hallmark of tumor development and progression (1). In this respect, thyroid neoplasms may epitomize an attractive prototype to explore because they incorporate different morphological and histopathological subtypes originating mostly from the same cell type, albeit with different grades of differentiation (2).

Germline mutations in *DICER1* and *DGCR8*, two of the main miRNA processing genes regulating miRNA maturation, are responsible for the DICER1 and familial multinodular goiter with schwannomatosis syndromes, respectively (3, 4). Patients with DICER1 syndrome usually exhibit truncating or frameshift *DICER1* alterations and may develop a plethora of tumors, including thyroid follicular nodular disease (FND) and well-differentiated thyroid carcinoma (WDTC) (5-9). *DGCR8* mutational carriers, on the other hand, often present with FND, whereas the true incidence of thyroid cancer within this syndrome is unknown (4, 10). In addition to germline alterations, subsets of sporadic thyroid tumors display recurrent hotspot mutations in these genes (11-17).

Somatic *DICER1* mutations have been reported across the entire spectrum of *RAS*-driven FTs, such as follicular thyroid adenoma (FTA), noninvasive follicular thyroid neoplasm with papillary-like nuclear features (NIFTP), invasive encapsulated follicular variant papillary thyroid carcinoma (IEFVPTC), follicular thyroid carcinoma (FTC), and rare cases of poorly differentiated thyroid carcinoma (PDTC) arising in pediatric/adolescent patients (8, 13, 18-21). *DGCR8* mutations on the other hand seem to aggregate in clinically troublesome cases and have been identified in subsets of FTC and PDTC with a particularly aggressive behavior (16, 17).

In all, *DICER1* mutations are believed to be more prevalent than *DGCR8* mutations in the sporadic setting. Both *DICER1* and *DGCR8* are postulated to exhibit tumor-suppressive functions, which is supported by the biallelic inactivation pattern observed in syndromic and sporadic cases. Specifically, biallelic *DICER1* mutations are common, and *DGCR8* mutations with loss of the remaining allele have been reported (6, 16, 22). For *DICER1* specifically, combinations of truncating mutations throughout the gene and hotspot mutations in the functionally important RNAse III region are commonly observed together. Interestingly, although truncating mutations may occur as both germline and somatic events, hotspot alterations are typically exclusively somatic (8).

In terms of functional connotations, analyses of global miRNA expression seem to support an aberrant profile for both *DICER1* and *DGCR8* mutants, which in turn may have consequences for gene regulation at the posttranscriptional level (16, 22, 23). Also, knock-down of *DICER1* mRNA in FTC cell lines promotes proliferation in vitro, thereby further strengthening the hypothesis of these miRNA regulators as tumor suppressor genes (15).

Despite these advances, most of what we know regarding the prevalence of *DICER1* and *DGCR8* mutations are data amassed from smaller, monoinstitutional characterizations, and other driver gene aberrations are not always accounted for. To counter this, FTs from two European high-volume thyroid cancer centers have been interrogated for *DICER1* and *DGCR8* hotspot mutations to establish the frequency of these genetic alterations. Additionally, to investigate the impact of these mutations, subsets of *DICER1* and *DGCR8* mutants were further analyzed for global miRNA output and exome sequenced to establish a potential relationship to other genetic drivers.

## Materials and Methods

**Figure 1.**
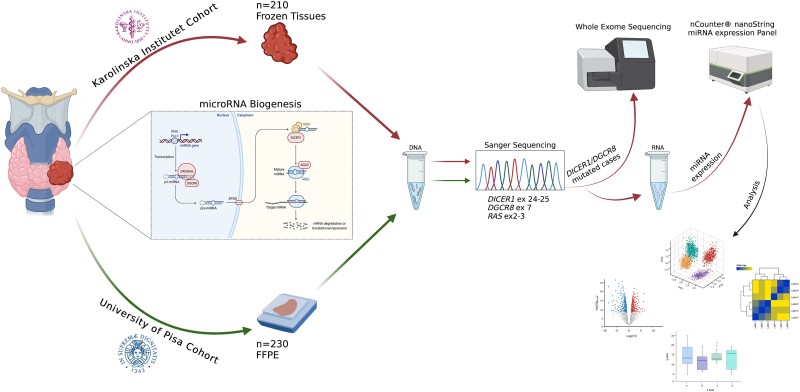
Summary of the tissue material and overall workflow applied in this study. A total of 440 thyroid lesions were included and Sanger sequenced for hotspot mutations in *DICER1*, *DGCR8,* and *RAS* genes. Karolinska Institutet cohort was solely based on fresh-frozen samples; thus, these samples were selected for subsequent whole-exome sequencing and global miRNA profiling. The figure was created with Biorender.com.

The workflow of the study is summarized in Fig. 1.

### Patient Cohort

Tumor samples were obtained from the Karolinska University Hospital, Stockholm, Sweden, and from the Unit of Surgical Pathology of the University of Pisa, Italy. Following approval of the institutional review boards, both institutions were queried for the diagnosis of FTs. This study included archived fresh-frozen tissues (Karolinska) and formalin-fixed and paraffin-embedded tissues (Pisa), totaling 40 FNDs, 127 FTAs, 48 NIFTPs, 1 follicular tumor of uncertain malignant potential (FT-UMP), 80 IEFVPTCs, 126 FTCs, 15 oncocytic thyroid carcinomas (OTC), and 3 differentiated high-grade thyroid carcinomas (DHGTCs). All cases were retrieved and reviewed to confirm the histological diagnosis by two different pathologists (C.C.J. and F.B.). Demographic information including age, sex, and nodule size were collected from each patient at the time of diagnosis, in addition to histopathologic results and tumor staging. In total, 440 FTs with these diagnoses were identified and collected. Single cases with previously established *DICER1* (n = 1) and *DGCR8* (n = 1) mutations were included for internal control of the Sanger sequencing procedure, in which the laboratory investigator was blinded of the genotype (15, 16). Constitutional DNA from normal thyroid tissue was later acquired for all cases with subsequently verified *DICER1* or *DGCR8* mutations, except for 2 pediatric cases in the Karolinska cohort for which normal tissue samples were not available.

### Nucleic Acids Isolation

For all included samples for which fresh-frozen tissues and formalin-fixed and paraffin-embedded were available, genomic DNA was extracted using DNeasy Blood and Tissue kit (Qiagen, Hilden, Germany) according to the manufacturer’s instructions, and concentration was evaluated using a spectrophotometer (NanoDrop 2000; Thermo Fisher Scientific, Waltham, MA).

Total RNA, including miRNAs, was isolated using miRNeasy Mini Kit (Qiagen) according to the manufacturer's instructions. The RNA concentration was assessed using a spectrophotometer (NanoDrop 2000; Thermo Fisher Scientific), and the quality control was assessed using the RNA 6000 nano kit (Bioanalyzer, Agilent, Santa Clara, CA). Only samples with a concentration ≥30 ng/μL, a ratio between the value of the absorbance at 260 nm and the absorbance at 280 nm was ≥1.8, and a ratio between the value of absorbance at 260 nm and that at 230 nm was ≥2 and with an RNA integrity number ≥ 7 were included for the analysis.

### Mutational Analysis

The hotspot mutational status of *DICER1* (exons 24-25), *DGCR8* (exon 7), and *N-H-KRAS* (exons 2-3) genes was investigated by direct sequencing (ABI 3730 DNA Analyzer, Applied Biosystems, Foster City, CA, USA) according to standard procedures in all 440 cases. Only *DICER1/DGCR8*-positive cases were further interrogated for the *PAX8::PPARG* fusion. All the primers used for sequencing are reported in Supplementary Table S1 (24). The paired normal tissues available from the contralateral lobe or the adjacent normal parenchyma of lesions harboring mutations in *DICER1* and *DGCR8* were used to confirm the somatic or germline nature of the variants according to current guidelines (25). All chromatograms were read in full, so amplified nucleotide sequences adjacent to hotspot regions were also assessed.

### Whole Exome Sequencing

Thirteen samples (8 *DICER1* or *DGCR8* mutated tumors and 5 corresponding normal tissues) were submitted for whole exome sequencing (Novogene, Cambridge, UK). For 2 *DICER1* mutant cases (T4 and T5), normal DNA was unavailable. For 1 *DGCR8* mutated tumor (T8), constitutional DNA was not submitted because this case was previously screened for germline involvement in a separate study (16). Briefly, genomic DNA of 8 tumor samples and 5 corresponding normal tissue samples was physically fragmented and prepared as libraries containing dual-indexed sequencing barcodes. The precapture libraries were enriched using Exome 2.0 Panel (Twist Bioscience) for coding regions and splice junction sites of 20 000 human genes. The postcapture libraries were sequenced using NovaSeq 6000 (Illumina). The sequence data were analyzed using a custom-developed bioinformatics pipeline that aligns sequence data to human genome (GRCh37/GRCh38) to perform variant calls and annotations. The pipeline quality control of the sequence data was performed to ensure the quality of the data and variant annotations were performed with Variant Effect Predictor tool.

### MicroRNA Profiling

A total of 24 samples were submitted for miRNA expression analysis, including 5 *DICER1* mutants, 2 *DGCR8* mutants, 15 wild-type tumors, and 2 normal thyroid samples. We also incorporated global miRNA profiling data acquired from a third *DGCR8* mutant case from an earlier publication using the same platform, bringing the total number of analyzed samples to 25. All wild-type samples were selected based on similar clinicopathologic features as the mutated cases.

The nCounter miRNA Expression Panel (nanoString Technologies, Seattle, WA, USA) was used for the analysis. The panel includes specific probes for 827 human miRNAs, 6 positive mRNA controls, 8 negative mRNA controls, 3 ligation-positive miRNA controls, 3 ligation-negative miRNA controls, 5 mRNA reference controls, and 5 spike-in controls.

Briefly, approximately 100 ng of total RNA from each fresh-frozen tissue sample were hybridized with probes containing unique barcodes and counted using the nCounter platform according to the manufacturer's instructions. Initial data quality control and data analysis were performed using nSolver v.4 (nanoString Technologies) and Rosalind (www.rosalind.bio). Data were normalized using the mean expression of the 100 most expressed miRNAs in the dataset. To select miRNA highly expressed among samples, the mean plus 2 SDs of negative control probes was used as threshold, and targets detected in less than 50% of samples were filtered out, as performed previously (26).

### Real-time RT-PCR

cDNA was synthesized using TaqMan MicroRNA Reverse Transcription Kit (Applied Biosystems) with assay-specific TaqMan primers, miR-135a-5p (hsa-miR-135a, 000460), miR-135b-5p (hsa-miR-135b, 002261), miR-181a-3p (hsa-miR-213, 000516), and the control assay RNU6B (001093) (Applied Biosystems) according to the manufacturer's instructions. Samples were run in QuantStudio1 Real-Time PCR System (Applied Biosystems) in triplicate with assay-specific TaqMan primers stated previously, and expression levels were calculated using the 2^−ΔΔCt^ method. Validation was only performed on *DICER1* mutated cases because they constituted the majority of the samples submitted for global miRNA analyses.

### Comparative Analyses in The Cancer Genome Atlas

Level 3 miRNA expression and clinical data of The Cancer Genome Atlas (TCGA) (27) on thyroid cancer were downloaded from Firehose (https://gdac.broadinstitute.org/). For downstream analyses, RPM log2 data of mature miRNA expression was used. Unsupervised hierarchical clustering was carried out using Euclidean distance, Ward clustering method, and following the procedures of heatmap3 R package v.1.1.9. The proportion of 5p and 3p miRNA strands were defined as the cumulative 5p and 3p miRNA levels in total. Differentially expressed miRNA (DEM) were computed by the Bayesian moderated t-statistics and following the procedures of the limma Bioconductor package v.3.56.2. *P* values were adjusted with the Benjamini-Hochberg method. A false discovery rate (FDR) below 0.05 was deemed significant. Venn diagrams were drawn to highlight overlapping DEM between *DICER1* and *DGCR8* mutant cases. Analyses and plots were generated in R environment (v.4.1.2, https://www.r-project.org/, last accessed June 22, 2023).

### Statistical Analyses

Differential expression analysis was calculated using nSolver v.4 and Rosalind softwares (nanoString Technologies). *P* values were adjusted with the Benjamini-Hochberg method. An FDR <0.05 was considered significant. Chi-square and Mann-Whitney *U* tests were used to compare clinicopathological features and a *P* value <.05 was considered significant. Statistical computations were performed using RStudio v12.0 and GraphPad Prism 9.5.1 (GraphPad Software, San Diego, CA, USA).

## Results

### Baseline Patient Characteristics

Patient and nodule characteristics are shown in detail in Table 1. The combined cohort from both institutions included a total of 440 patients with FTs, of which 38% were classified as benign (40 FND, 127 FTA), 11% low-risk (48 NIFTP, 1 follicular tumor of uncertain malignant potential), and 51% malignant (80 EFVPTC, 126 FTC, 15 oncocytic thyroid carcinomas, and 3 DHGTC). The mean age was 48.8 ± 16.7 years, and 72.3% (n = 319) were female. Only 6 cases (1.3%) were considered pediatric/adolescent patients (ranged in age from 10 to 18 years).

**Table 1.** Clinicopathological characteristics of 440 follicular-patterned thyroid nodules included in the cohort

	Benign, n = 167 (38%)		Low-Risk, n = 49 (11%)		Malignant, n = 224 (51%)				
	FND n = 40	FTA n = 127	NIFTP n = 48	FT-UMP n = 1	EFVPTC n = 80	FTC n = 126	OTC n = 15	DHGTC n = 3	All samples n = 440
**Sex**									
Female	32	88	37	1	56	94	8	3	319
Male	8	39	11	/	23	33	7	/	121
**Age, y, mean ± SD**	58.2 ± 15	48.5 ± 15	47.3 ± 12.7	49	48.1 ± 15.7	53.4 ± 19.3	50.2 ± 17.3	51 ± 22.6	48.8 ± 16.7
**Nodule size, cm, mean ± SD**	NA	3.2 ± 1.5	2.8 ± 1.5	7	2.6 ± 1.3	4 ± 2.2	3.9 ± 1.9	8.4 ± 7	3.3 ± 1.8
**Tumor invasiveness**									
Minimally invasive	/	/	/	/	50	57	8	n.a.	115
Widely invasive	/	/	/	/	19	34	5	n.a.	58
Angioinvasive	/	/	/	/	4	36	2	n.a.	42
Infiltrative	/	/	/	/	7	/	/	n.a.	7

Abbreviations: DHGTC, differentiated high-grade thyroid carcinoma; EFVPTC, encapsulated follicular variant papillary thyroid carcinoma; FND, follicular nodular disease; FTA, follicular thyroid adenoma; FTC, follicular thyroid carcinoma; FT-UMP, follicular tumor of uncertain malignant potential; NA, not available; NIFTP, noninvasive follicular thyroid neoplasm with papillary-like nuclear features; OTC, oncocytic thyroid carcinoma.

### *DICER1* and *DGCR8* Mutation Frequencies in Follicular-patterned Thyroid Lesions

**Figure 2.**
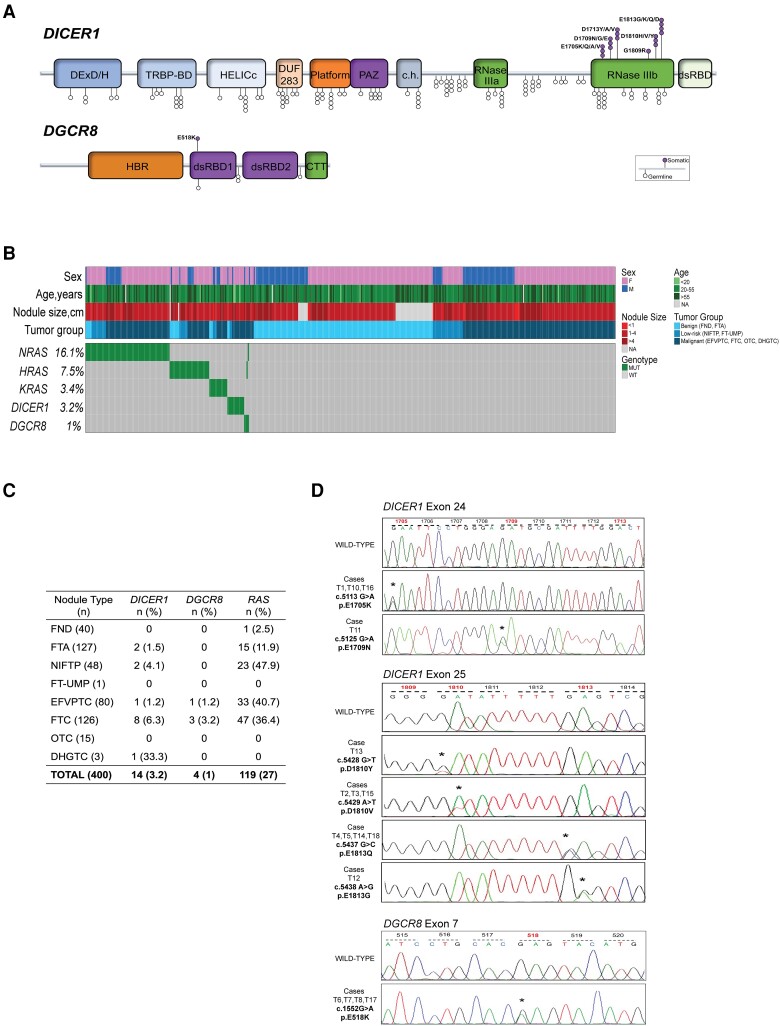
Breakdown of *DICER1* and *DGCR8* mutational analysis. (A) Lollipop plot highlighting the 6 known somatic hotspot mutations in *DICER1* and 1 in *DGCR8*. Somatic hotspots are illustrated at the top, whereas common germline variants are shown at the bottom of the figure. (B) Oncoprint plot depicting Sanger sequencing results from 440 follicular-patterned thyroid nodules from the bi-institutional cohort. Each column represents 1 sample, and each row represents a gene. (C) Summary of the mutations discovered in the cohort stratified into unique diagnostic entities. (D) Representative chromatograms obtained by Sanger sequencing analysis showing somatic missense mutations in *DICER1* and *DGCR8* (chromatogram for T17 carrying *DICER1* E1813K was not available).

To establish an overall mutational frequency, a total of 440 consecutive FTs from the two institutions were interrogated for hotspot mutations in *DICER1* and *DGCR8* genes using Sanger sequencing. We identified 18 (4.1%) cases carrying hotspot mutations in either *DICER1* or *DGCR8*, and these events were mutually exclusive. Fourteen (3.2%) cases showed hotspot mutations within the RNase IIIb domain of the *DICER1* gene, including E1705K (n = 2), and D1709N (n = 1) (exon 24); D1810Y (n = 1), D1810V (n = 4), E1813K (n = 1), E1813G (n = 1), and E1813Q (n = 4) (exon 25). Four (1%) cases showed E518K mutations of the *DGCR8* gene, whereas 1 case carried an L516P mutation. The latter alteration is not a previously reported hotspot mutation but was identified when reading through the entire chromatogram. For all the positive cases, including the *DGCR8* L516P mutated case, in which constitutional DNA was available (n = 16), the germline/somatic status was tested, showing a somatic origin of all mutations. Point mutations within the *RAS* gene family were also interrogated by examining codons 12, 13, and 61 for *N-H-KRAS*. Collectively, 119 (27%) cases showed *RAS* hotspot mutations, including *NRAS* (n = 71, 16.1%), *HRAS* (n = 33, 7.5%), and *KRAS* (n = 15, 3.4%). Two cases, both *DGCR8* mutated, showed *N*- and *HRAS* mutations, respectively, whereas the remaining cases did not show any overlap. Results are detailed in Fig. 2.

### Clinical Characteristics of *DICER1* and *DGCR8* Mutated Cases

The 18 *DICER1*/*DGCR8* mutated cases included 15 females (83%) with a mean age of 41 ± 19 years (median, 36) and a mean nodule size of 4.3 ± 3.4 cm (median, 3.5). Histopathological data were available for all these patients, but only 8 had clinical follow-up information available of >10 years. The whole spectrum of benign, low-risk, and malignant lesions harbored *DICER1* mutations, including 2 FTAs, 2 NIFTPs, 1 angioinvasive EFVPTC, 4 minimally invasive FTCs, 4 angioinvasive FTCs, and 1 DHGTC, whereas 1 minimally invasive EFVPTC and 3 widely invasive FTCs harbored a *DGCR8* mutation (Fig. 2C). Cases harboring *DICER1* mutations (T1-T5) with a follow-up of >10 years were all alive without recurrent disease at the time of this study. Among all 14 *DICER1* mutated cases, 12 (86%) were female and only 2 were pediatric patients (aged 10 and 17 years, respectively), whereas the remaining 12 patients harboring *DICER1* mutations had an age ranging from 24 to 77 years. A significant association between *DICER1* mutations and younger age at surgery (*P* = .004) was found.

Of 4 cases harboring *DGCR8* mutations, 2 (T6 and T7) showed recurrent disease with metastatic spread to lungs and bone, and both died of the disease 10 and 12 years after the diagnosis, respectively. The third case (T8) presented elevated thyroglobulin levels at the last examination but no structural evidence of relapse or metastases. For the fourth case (T17), no follow-up information was available. Among the 4 *DGCR8* mutated cases, 3 (75%) were female and showed a significant association with the larger nodule size (*P* = .009) compared with the wild-type cases. Histopathologic characteristics of the positive cases are summarized in Table 2.

**Table 2.** Clinical characteristics of all *DICER1* and *DGCR8* mutated cases

Case ID	Sex	Age at surgery, y	Nodule size, cm	Tumor type	pTNM	Gene mutation	*RAS*	*PAX8::PPARG*
**T1**	F	24	3.5	MIFTC	pT2Nx	*DICER1* c.5113 G > A p.E1705K	WT	WT
**T2**	F	29	2.4	FTA	NA	*DICER1* c.5429 A > T p.D1810V	WT	WT
**T3**	F	47	3.5	MIFTC	pT2Nx	*DICER1* c.5429 A > T p.D1810V	WT	WT
**T4**	F	10	4.7	EAIFTC	pT3aNx	*DICER1* c.5437 G > C p.E1813Q	WT	WT
**T5**	F	17	5	EAIFTC	pT3aNx	*DICER1* c.5437 G > C p.E1813Q	WT	WT
**T6**	M	66	8	WIFTC	pT3aNx	*DGCR8* c.1552 G > A p.E518K	WT	WT
**T7**	F	74	NA	WIFTC	NA	*DGCR8* c.1552 G > A p.E518K	WT	WT
**T8**	F	31	5	WIFTC	pT3	*DGCR8* c.1552 G > A p.E518K	HRAS c.182 A > G p.Q61R	WT
**T9**	F	50	1.5	NIFTP	/	*DICER1* c.5125 G > A p.D1709N	WT	WT
**T10**	F	35	3.5	NIFTP	/	*DICER1* c.5113 G > A p.E1705K	WT	WT
**T11**	F	18	1.6	EAIFTC	pT1bNxMx	*DICER1* c.5125 G > A p.E1709N	WT	WT
**T12**	M	77	16	DHGTC	pT3aNxM1	*DICER1* c.5438 A > G p.E1813G	WT	WT
**T13**	F	33	1.2	MIFTC	pT1bNxMx	*DICER1* c.5428 G > T p.D1810Y	WT	WT
**T14**	F	47	1.6	MIFTC	pT1bNxMx	*DICER1* c.5437 G > C p.E1813Q	WT	WT
**T15**	F	54	3	EAIFTC	pT2N0Mx	*DICER1* c.5429 A > T p.D1810V	WT	WT
**T16**	F	37	3.9	AIEFVPTC	pT2N0Mx	*DICER1* c.5113 G > A p.E1705K	WT	WT
**T17**	F	54	5	MIEFVPTC	pT3aNxMx	DGCR8 c.1552 G > A p.E518K	NRAS c.182 A > C p.Q61R	WT
**T18**	M	35	3.4	FTA	/	*DICER1* c.5437 G > A p.E1813Q	WT	WT

Abbreviations: AEIFVPTC, angioinvasive encapsulated follicular variant papillary thyroid carcinoma; AIFTC, angioinvasive follicular thyroid carcinoma; DHGTC, differentiated high-grade thyroid carcinoma; EAIFVPTC, encapsulated angioinvasive follicular variant papillary thyroid carcinoma; MIEFVPTC, minimally invasive encapsulated follicular variant papillary thyroid carcinoma; MIFTC, minimally invasive follicular thyroid carcinoma; NA, not available; WIFTC, widely invasive follicular thyroid carcinoma; WT, wild-type.

### *DICER1* Mutated Thyroid Lesions Lack Mutations in Established Thyroid Cancer Drivers, Whereas *DGCR8* Mutations May Coexist With *RAS* Aberrations

All the sequencing quality parameters are listed in Supplementary Table S2 (24).

The mutational landscape of all *DICER1* and *DGCR8* mutated found in the Karolinska cohort (T1-T8) was performed using whole-exome sequencing to identify additional driver mutations and verify the somatic origin of the alterations whenever possible.

First, to determine whether *DICER1* and *DGCR8* mutations were truly somatic or germline, the paired normal tissues of 5 cases were also submitted for analysis, the results of which are consistent with a somatic origin of the mutations for the informative cases.

All positive cases yielded the same *DICER1* and *DGCR8* missense mutation variants detected by Sanger sequencing. All 5 *DICER1* mutated cases (T1-T5) also exhibited a second, non-hotspot *DICER1* variant (truncating or splicing) in addition to the missense known variant.

**Figure 3.**
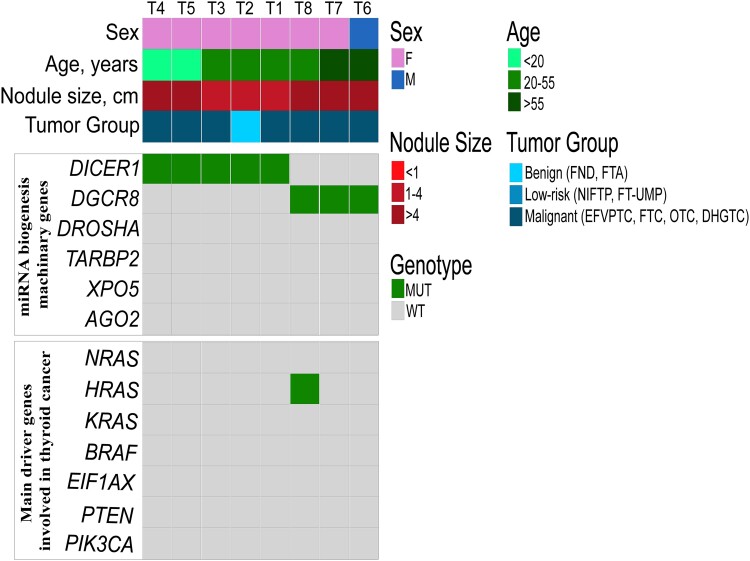
Oncoprint plot showing the molecular landscape obtained by whole-exome sequencing analysis of 5 *DICER1* and 3 *DGCR8* mutated cases found in the Karolinska cohort highlighting well-known thyroid-related genes and other genes involved in the miRNA biogenesis machinery. Besides a single *HRAS* mutation found in T8, all other samples were wild-type for additional thyroid-related genes as well as additional genes involved in the miRNA machinery.

Next, we mainly focused on the genetic mutations of established thyroid cancer driver genes or thyroid cancer progression. None of the 8 tumors showed alterations in known hotspots of thyroid-related genes such as *BRAF, EIF1AX, PIK3CA, PTEN, TP53, CDKNA2,* and *RBM10* genes (Supplementary Table S2) (24). An *HRAS* Q61R mutation was confirmed in the case harboring the *DGCR8* E518K mutation (T8). Moreover, no additional mutations were found in the other genes involved in the miRNA processing machinery, such as *TARBP2, DROSHA, XPO5,* or *AGO2* (Fig. 3).

To further investigate the real impact of *DICER1* and *DGCR8* mutations on these tumors, all positive samples from both institutions were also interrogated for *PAX8::PPARG* fusions using RT-PCR analysis; none harbored this alteration (Table 2).

### Impact of *DICER1* and *DGCR8* Mutations on Global miRNA Expression

**Figure 4.**
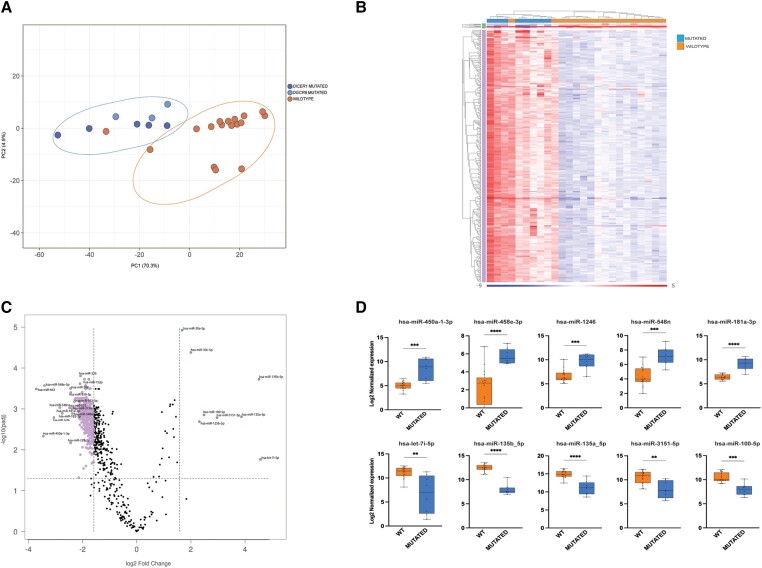
miRNA dysregulation in thyroid nodules harboring somatic hotspot mutations in *DICER1* and *DGCR8* genes. (A) Principal component analysis (PCA) based on *DICER1* (n = 5) and *DGCR8* (n = 3) mutated cases found in the Karolinska cohort and 17 wild-type cases (matched for age, sex, and diagnoses) (B) Heatmap of the 427 differentially expressed miRNAs between wild-type and mutated cases. All *DICER1/DGCR8* mutated cases clustered together in a separate branch (left part of the heatmap) (C) Volcano plot showing the most dysregulated miRNAs between mutated and wild-type cases. (D) Top 5 up- and top 5 down-regulated miRNAs in mutated compared with wild-type cases. The first row shows 5 miRNAs up-regulated in mutated cases compared with wild-type; the second row shows 5 miRNAs down-regulated in mutated compared with wild-type.

We next sought to investigate whether *DICER1* and *DGCR8* mutations might have an influence on miRNA biogenesis and expression. We first used a principal component analysis (PCA) to carry out an unsupervised investigation of the relationship between tumor genotype and global miRNA expression. As seen in Fig. 4A, PCA revealed that all the *DICER1*/*DGCR8* positive cases clustered together, showing a common and unique miRNA profile driven by mutations and independent of histology. One widely invasive FTC wild-type for *DICER1* and *DGCR8* hotspot mutations clearly clustered together with the mutated samples on both PCA plot and miRNA heatmap (Fig. 4A and 4B, respectively). This sample was submitted for further molecular analysis, using a next-generation sequencing panel used in clinical routine that covers the whole *DICER1* gene (22). No other mutations were found in *DICER1* (data not shown). Furthermore, we interrogated the same case for the most common hotspot mutations in *DROSHA* gene, but no alterations were found. The clustering of this single sample thus remains elusive but may involve epigenetic dysregulation of miRNA-related genes, previously shown for FTCs (15). Moreover, a second *DICER1/DGCR8* wild-type case appears to cluster with the mutated group in Fig. 4B, but this was not clearly reflected in the PCA plot. Given the ambiguity in its association with the mutated cluster in terms of miRNA dysregulations, we opted not to screen this tumor for additional miRNA-regulating gene mutations.

Next, a differential miRNA expression analysis was performed to explore differences between *DICER1/DGCR8* mutated and wild-type cases. Setting a cutoff FDR of 0.05 with a fold change ≥3, a total of 427 DEMs were found between wild-type and mutated cases, as shown in Fig. 4B and 4C. Among these, we searched for individual miRNAs that had the highest and lowest levels of expression in wild-type versus mutated. The top 5 upregulated and downregulated miRNAs were selected and are shown in Fig. 4D. Five miRNAs (miR-450a-1-3p, miR-548e-3p, miR-1246, miR-548n, and miR-181a-3p) were overexpressed in mutated cases, whereas 5 additional miRNAs (let-7i-5p, miR-135b-5p, miR135a-5p, miR3151-5p, and miR-100-5p) were most consistently downregulated in mutated cases. Most have been previously reported to be deregulated in thyroid cancer (18, 28, 29). Using the same cutoff, no significant differences were found when we compared *DICER1* with *DGCR8* mutated cases only.

**Figure 5.**
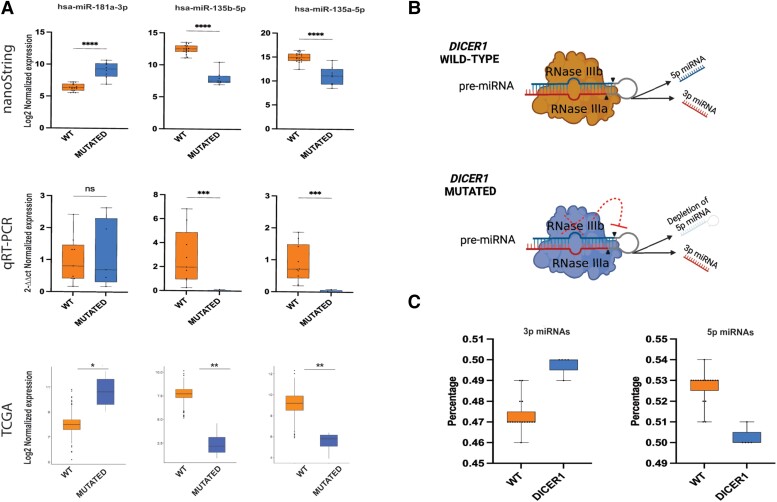
Extended miRNA expression validation in thyroid nodules with *DICER1*/*DGCR8* mutations. (A) Top: expression levels of 3 selected miRNAs using global miRNA profiling via nanoString; middle: relative expression levels of the same 3 selected miRNAs validated experimentally by quantitative RT-PCR; bottom: expression levels of the same 3 selected miRNAs extracted from TCGA data. (B) Disabling mutations in RNase III domains of *DICER1* lead to 5p miRNA depletion. Schematic representation of the mechanism by which DICER1 cleaves pre-miRNA in wild-type and mutated conditions; hotspot mutations in the RNase IIIb domain lead to a significant reduction of 5p strand miRNAs in *DICER1* mutants compared with wild-type cases. The figure was created with Biorender.com. (C) Remarkable difference in 5p:3p strand ratio between *DICER*1 mutants and wild-type cases was observed in the Karolinska cohort.

Predictably, all the *DICER1* mutated cases displayed a reduced expression of 5p miRNAs and an increased level of 3p miRNAs compared with wild-type cases, supporting the hypothesis that these mutations determine an evident imbalance 5p:3p miRNAs (Fig. 5B-C). These results are clearly consistent with other studies (18, 23).

Furthermore, the DEMs in wild-type vs mutated cases have been used for pathway enrichment analysis on the DIANA-miRPath v3.0 tool, and more than 50 pathways resulted significantly enriched. In Table 3, the top 20 pathways enriched, ranked by ascending *P* values, are detailed.

**Table 3.** List of the top 20 significantly enriched pathways controlled by the miRNAs deregulated in *DICER1/DGCR8* mutant vs wild-type

	Pathways (KEGG)	Adjusted *P*	Number of target genes	Number of miRNAs
1	Protein processing in endoplasmic reticulum	8.66e-14	125	53
2	Proteoglycans in cancer	1.13e-12	134	54
3	Viral carcinogenesis	7.29e-11	126	58
4	Adherens junction	4.06e-09	58	48
5	Hippo signaling pathway	4.06e-09	98	55
6	Prostate cancer	3.30e-07	68	49
7	Pancreatic cancer	4.23e-07	49	48
8	Colorectal cancer	8.67e-07	49	47
9	Bacterial invasion of epithelial cells	2.23e-06	57	50
10	Ubiquitin mediated proteolysis	2.23e-06	95	52
11	Cell cycle	2.67e-06	88	56
12	Central carbon metabolism in cancer	3.83e-06	50	43
13	Renal cell carcinoma	5.61e-06	49	42
14	Lysine degradation	6.24e-06	32	46
15	Oocyte meiosis	6.68e-06	76	50
16	TGF-β signaling pathway	9.58e-06	53	48
17	Endocytosis	9.58e-06	134	50
18	Small cell lung cancer	1.23e-05	63	47
19	p53 signaling pathway	1.23e-05	52	52
20	HIF-1 signaling pathway	1.68e-05	74	47

Abbreviations: KEGG, Kyoto Encyclopedia of Genes and Genomes; miRNA, micro-RNA.

### Data Validation Using the TCGA Database

To further validate our results, all *DICER1* and *DGCR8* mutant cases with clinical parameters were retrieved from the TCGA database of PTCs (Supplementary Table S3) (24). First, a clustering analysis was performed to detect differences in terms of miRNA expression profiles. All mutated cases clustered together on a separate branch. One exception was the only case with a *DICER1* mutation that was not located in the RNase IIIb domain (Supplementary Fig. S1) (24).

As expected, the cumulative proportion of 3p strand miRNAs was higher in the 2 tumors harboring *DICER1* RNase IIIb mutations (Supplementary Fig. S2) (24); hence, only these 2 cases were considered as *DICER1* mutated in the following analyses. Cases harboring *DGCR8* E518K showed no consistent loss of 5p miRNAs.

Differential expression analysis highlighted 106 and 72 DEMs in *DICER1* and *DGCR8* mutant tumors, respectively. Thirty-seven miRNAs were commonly deregulated (Supplementary Fig. S3A) (24). A remarkable effect of miRNA strand was observed in *DICER1* mutant only, whereas 3p miRNAs were upregulated; the expression of 5p miRNA was suppressed (Supplementary Fig. S3B and 3C) (24). Looking at DEMs in *DGCR8*-mutant tumors, 10 miRNA pairs (ie, 3p and 5p miRNAs originating from the same precursor) were deregulated in the same direction (ie, either both up- or downregulated). In detail, 4 miRNA pairs were suppressed (ie, miR-141, miR-29c, miR-708, miR-30d), whereas the expression levels of 6 miRNA pairs were enhanced (ie, miR-145, miR-143, miR-378a, miR-126, miR-140, miR-29b-2).

Among the top 5 up- and downregulated DEMs of the nanoString analysis performed in our institutional cases, 3 miRNAs (miR-135b-5p, miR135a-5p, and miR-181a-3p) were consistently deregulated also in the TCGA series. These miRNAs were further investigated by quantitative RT-PCR in 5 *DICER1-*positive cases and 10 wild-type cases with similar clinicopathologic features as the mutated cases. A reliable concordance between results using different techniques was found (Fig. 5A). Only the miRNA miR-181a-3p did not reach statistical significance using this method.

## Discussion

With the recent identification of recurrent *DICER1* and *DGCR8* hotspot mutations in subsets of WDTCs (16, 20), the interest in genes involved in miRNA processing machinery has grown among researchers. However, the question arises whether these alterations are independent driver gene alterations or late events unrelated to tumorigenesis per se. Our integrated analysis reports the prevalence and spectrum of *DICER1* and *DGCR8* mutations in an unselected, bi-institutional cohort of FTs and shows how these alterations clearly impact the global miRNA expression status. Furthermore, we strengthened the robustness of our data by comparing our findings with data from the TCGA cases in terms of clinical parameters and influence on miRNA expression profile.

In the past few years, several studies reported *DICER1* biallelic inactivation at the somatic level, predominantly in WDTC, lacking genetic alterations of established thyroid cancer driver genes, strongly suggesting that *DICER1* mutations may trigger the onset of specific subgroups of thyroid tumors (20). Somatic mutations in *DICER1* RNase IIIb hotspots have been shown in a wide variety of *RAS*-like lesions, ranging from benign FTA (23), NIFTP (30), to malignant FTC (22) and FVPTCs (12), highlighting an association to early- and adult-onset thyroid lesions with a favorable prognosis (31, 32). By contrast, Chernock and collaborators recently demonstrated how childhood- and adolescent-onset PDTC are genetically distinct from adult-onset PDTC in that they were strongly associated with *DICER1* mutations, suggesting that the clinically aggressive behavior of PDTC contrasts sharply with the indolent nature of most thyroid lesions driven by *DICER1* mutations reported to date (21).

In the present study, 3.2% of the cohort cases harbored *DICER1* hotspot mutations, occurring in FTA (2/127), NIFTP (2/48), EFVPTC (1/80), FTC (8/126), and DHGTC (1/3). Of these, 5 cases were found to harbor a second, non-hotspot *DICER1* variant in addition to the primary missense mutation when analyzed via WES. Among the 14 mutated cases, the p.E1813Q missense mutation occurs in 2 pediatric patients (aged 10 and 17 years, respectively), whereas the remaining patients ranged in age from 24 to 77 years. Interestingly, a recent study reported that the frequency of *DICER1* hotspot mutations in thyroid nodules with indeterminate cytology was 1.4%, occurring in a proportion of adult patients and appearing mutually exclusive with alterations in other thyroid cancer-related genes (20). However, it is worth mentioning that these data, including the current study, include hotspot alterations only, and therefore the true prevalence of *DICER1* mutated cases (including non-RNase IIIb region mutations) remains to be established.

In our cohort, patients with *DICER1* mutated lesions were younger than *DICER1* wild-type cases, corroborating previous observations (13, 14, 22). Although the *DICER1*-mutated patient group as a whole skewed toward a younger demographic, it is crucial to note that all mutations identified in this study that could be tested in constitutional DNA were of somatic origin. However, T4 and T5 originate from pediatric patients (10-year-old and 17-year-old females, respectively) with *DICER1*-mutated encapsulated angioinvasive FTC. For these cases, constitutional tissue was unavailable for sequencing, leaving the germline *DICER1* status unknown. Despite this, we chose to include them in the whole-exome sequencing analysis because potential biallelic inactivation of *DICER1* would support its role as a driving force behind tumor development in these patients. Both cases exhibit no recurrences or indications of other tumor manifestations and exhibit negative family history concerning lesions related to DICER1 syndrome.

A pertinent question emerges: Should every patient with *DICER1* mutated thyroid nodules, identified through molecular workup, undergo clinical genetics evaluation to rule out DICER1 syndrome? At present, neither of our institutions authoring this study perform clinical routine reflex sequencing of *DICER1* and *DGCR8*. Our data, when considered alongside previous observations, suggest that the majority of *DICER1* mutations in thyroid tumors are indeed somatic, even in cases of biallelic mutations. However, it is imperative to factor in elements such as family medical history, the presence of other tumor manifestations, and the overall disease presentation when making this determination. Certain studies have identified subsets of patients with germline *DICER1* involvement, even in cases lacking a positive family history and other manifestations beyond thyroid follicular nodular disease (33). Consequently, considering the potential clinical benefit, it might be worthwhile to screen patients with identified *DICER1* variants in tumor tissue for constitutional involvement. Given the infrequency of these mutations in routine clinical practice, the overall cost associated with clinical genetics counseling and germline DNA sequencing is likely to be minimal in comparison to the potential benefits of identifying a patient with a syndromic predisposition. Therefore, the threshold for initiating germline DNA investigations should be set relatively low. Nevertheless, our study is underpowered in terms of cases analyzed for germline involvement, which prevents us from providing comprehensive advice on genetic counseling. Future studies on this topic could eventually provide clarity regarding the overall frequency of these genetic alterations in thyroid nodules and might be helpful in constructing guidelines for clinical practice. It must also be stressed that we focused on hotspot alterations only, meaning that the entire genes were not fully interrogated in this study.

For *DGCR8* mutations, the number of published cases is limited. Although there may be an association with aggressive biological behavior, certain patients with this mutation occurring in the germline develop thyroid follicular nodular disease rather than thyroid cancer (4). Consequently, the established value of identifying this mutation in somatic screening remains uncertain.

Regarding the *DGCR8* gene, the specific E518K variant causes multinodular goiter with schwannomatosis (4), and somatic loss of heterozygosity has been shown as a second event in tumor tissues from these patients. The same combination has also been shown to act as driver event in Wilms tumor, thus strongly advocating for a tumor suppressor function (34). Recently, our group reported somatic E518K mutations with concurrent loss of heterozygosity in FTCs, showing how this mutation potentially influences the aggressive behavior of these tumors (16). Another study reported the same mutation in a single PDTC (17). We identified 4 cases harboring the E518K mutation (including 1 previously published case), of which 3 were detected in aggressive FTCs and 1 in an IEFVPCT, suggesting that E518K variant could be specific for high-risk malignancies. However, because of the limited number of published cases with this alteration, further investigations are needed before a true causality between this genotype and clinical attributes can be verified. This is particularly important because subsets of cases with germline *DGRC8* mutations only seem to develop thyroid follicular nodular disease.

Furthermore, we provided evidence of how miRNA expression profiles vary in *DICER1/DGCR8* mutated compared with wild-type tumors. The impact of these mutations at the miRNA expression level is explained by a common and unique molecular signature on mutated cases, pointing toward the possible variation in miRNA signatures depending on the mutational status of these 2 genes independently of histology. However, the mechanistic reason for the altered miRNA expression may vary between *DICER1* and *DGCR8* mutants, as 3p:5p miRNA imbalance is typical of *DICER1* mutations, whereas dysregulated processing of specific precursors is the hallmark of *DCGR8* mutations. Among the DEMs, we searched for individual miRNAs that had the highest and lowest levels of expression in wild-type vs mutated. We particularly focused on those that were already described to have a key role in thyroid lesions. In particular, miR-181a-3p was previously described as deregulated in NIFTP and IFVPTC and associated with adverse outcomes in patients with EFVPTC (28, 29). miR-135a-5p and miR-135b-5p were recently described as downregulated in *DICER1* mutated pediatric thyroid cancer cases (18). These 2 miRNAs have also been associated with tumor suppressor function in PTC (35). Finally, the present investigation supports the currently held dogma: *DICER1* mutations strongly impair miRNA biogenesis. Predictably, the imbalance 5p:3p in all those cases carrying *DICER1* RNase IIIb hotspot mutations was found, in line with previous studies (18, 23, 36).

The findings of miRNA dysregulation in *DICER1* and *DGCR8* mutants were consistently supported by the results obtained by analyzing TCGA data from PTCs, suggesting that the mutational effect on miRNA profiles is apparent irrespective of tumor type. This strongly supports our hypothesis that the mutational status of *DICER1* and *DGCR8* rather than tumor histotype determines the global change in miRNA expression. Moreover, a sharp detection of imbalance 5p:3p miRNAs between *DICER1* mutated cases compared with wild-type cases was demonstrated, suggesting a similar modus operandi also for PTCs.

In conclusion, we provide an overall mutational frequency of *DICER1* and *DGCR8* mutations in a bi-institutional cohort of thyroid nodules, showing that these mutations are rare events in FTs. Furthermore, we corroborated that *DICER1* mutations may have independent abilities to trigger the onset of specific subgroups of thyroid lesions, whereas *DGCR8* mutations may coexist with bona fide thyroid drivers but may stimulate progression because this genetic event was congregated in tumors with aggressive behavior and poor outcome. Mutations in these 2 genes have a clear impact on global miRNA expression, strongly implying that the mutations cause dysregulation of the miRNA machinery. Future investigations of dysregulated miRNAs in *DICER1* and *DGCR8* mutated tumors may provide important clues for thyroid tumor initiation and progression.

## Data Availability

Some or all datasets generated during and/or analyzed during the current study are not publicly available but are available from the corresponding author on reasonable request.

## Abbreviations

DEM: differentially expressed micro-RNA
DHGTC: differentiated high-grade thyroid carcinoma
FDR: false discovery rate
FND: follicular nodular disease
FTA: follicular thyroid adenoma
FTC: follicular thyroid carcinoma
IEFVPTC: invasive encapsulated follicular variant papillary thyroid carcinoma
miRNA: micro-RNA
NIFTP: noninvasive follicular thyroid neoplasm with papillary-like nuclear features
PCA: principal component analysis
PDTC: poorly differentiated thyroid carcinoma
TCGA: The Cancer Genome Atlas
WDTC: well-differentiated thyroid carcinoma
